# Synergistic Effects of Partial Substitution of Sludge with Cattle Manure and Straw on Soil Improvement and *Pinus sylvestris* var. *mongolica* Growth in Horqin Sandy Land, China

**DOI:** 10.3390/plants14132067

**Published:** 2025-07-06

**Authors:** Dan Su, Meiqi Zhang, Yao Chang, Jie Bai, Guiyan Ai, Yanhui Peng, Zhongyi Pang, Xuekai Sun

**Affiliations:** 1 College of Environmental Science, Liaoning University, Shenyang 110036, China; iamsudan@126.com (D.S.); zhangmeiqi00@126.com (M.Z.); changy712@163.com (Y.C.); 2 CAS Key Laboratory of Forest Ecology and Silviculture, Institute of Applied Ecology, Chinese Academy of Sciences, Shenyang 110016, China; awwagy@126.com; 3 State-Owned Xinmin City Machinery Forest Farm, Shenyang 110300, China; xmjxlcpyh@163.com (Y.P.); 13478192389@163.com (Z.P.)

**Keywords:** soil amendments, *Pinus sylvestris* var. *mongolica*, sandy land, nutrient-poor soil, synergistic effects

## Abstract

Afforestation with *Pinus sylvestris* var. *mongolica* in northern China is hindered by soil degradation. This study evaluated a ternary amendment combining sewage sludge (SS), cattle manure (CM), and maize straw (MS) to rehabilitate degraded sandy soils in the Horqin Sandy Land. Five treatments were tested: control (CK), SS (T1), SS + CM (T2), SS+MS (T3), and SS + CM + MS (T4). The ternary amendment (T4) achieved optimal outcomes: soil pH decreased from 8.02 to 7.79, organic carbon increased 2.5–fold, and total nitrogen (127%) and phosphorus (87.5%) were enhanced compared to CK. *Pinus sylvestris* exhibited a 65.6% greater basal diameter and 29.5% height increase under T4, while heavy metal concentrations (Cd: −54.6%, Cu: −35.1%, Pb: −12.2% and Zn: −27.6%) were reduced. These findings highlight a synergistic waste valorization strategy for dryland afforestation, balancing soil fertility improvement with ecological safety. Future studies should prioritize long-term microbial community dynamics and field-scale validation.

## 1. Introduction

*Pinus sylvestris* var. *mongolica*, a drought-tolerant conifer, was introduced to the Three-North regions (Northeast, North, and Northwest China) during the mid-20th century to combat desertification. Currently spanning over 8.0 × 10^5^ hectares, this species serves as a critical ecological barrier against sand encroachment and soil erosion [1,2]. However, its long-term sustainability in arid and semi-arid zones, particularly in the Horqin Sandy Land—one of China’s severely desertified regions—is increasingly compromised by nutrient-depleted soils with inherently low organic matter (<1.5%), poor water retention, and accelerated degradation from anthropogenic pressures such as overgrazing and mining [3,4]. These conditions restrict vegetation restoration and ecosystem resilience.

To address these challenges, organic amendments including sewage sludge (SS), cattle manure (CM), and maize straw (MS) have emerged as promising rehabilitation tools. SS, a nutrient-rich byproduct of wastewater treatment, provides substantial organic carbon (39.32 g·kg^−1^) and macronutrients but poses risks of heavy metal accumulation (e.g., Cd, Pb) and pathogen persistence [5,6]. In contrast, CM enhances soil aggregation and supplies potassium (K), calcium (Ca), and magnesium (Mg) without significant contamination risks [7], while MS stabilizes soil carbon and stimulates microbial activity via lignin–cellulose decomposition [8]. Recent advancements in hybrid organic amendments, such as biochar-compost blends, have demonstrated enhanced remediation potential for sandy soils. For instance, Khan et al. [9] demonstrated that biochar–compost blends (Biochar is a carbon-rich pyrogenic substance derived from biomass resources such as agricultural waste, wood waste, forest residues, and food waste through pyrolysis) soils through enhanced increased soil organic carbon by 2.8–fold and water retention by 30% in arid sandy humic acid complexation. Furthermore, microbial inoculants (e.g., *Pseudomonas fluorescens*, *Trichoderma harzianum*) have emerged as a complementary strategy to enhance nutrient cycling efficiency in sandy ecosystems. Studies indicate that microbial amendments (such as *Enterobacter ludwigii San8* and *Rhizobium* sp. *G-01*) increase urease and acid phosphatase activities by 40–60% through stimulation of nitrogen-fixing and phosphate-solubilizing functional guilds, stabilize soil aggregates via extracellular polymeric substances (EPS), and alleviate plant drought stress through exopolysaccharide secretion [10,11]. Despite their individual merits, the isolated application of these amendments fails to address the multifactorial limitations of sandy soils, such as imbalanced nutrient stoichiometry and transient organic matter retention. Recent studies propose that partial substitution of SS with CM and MS could synergistically optimize soil rehabilitation by integrating their complementary properties: CM mitigates sludge-derived alkalinity through organic acid release, while MS improves porosity and microbial diversity [12,13]. However, mechanistic insights into these interactions—particularly their effects on soil–plant feedbacks in nutrient-poor sandy ecosystems—remain poorly understood.

Thus, more works are needed to understand how ternary amendments (SS + CM + MS) application balance soil fertility enhancement with heavy metal (Cu, Zn, Cd, and Pb) pollution risk mitigation and quantify their impacts on *Pinus sylvestris* var. *mongolica* performance under field conditions. In this study, we have hypothesized that integrated SS–CM–MS amendments will (1) improve soil physicochemical properties (pH, soil moisture, soil organic carbon, total nitrogen, and total phosphorus), (2) enhance *Pinus sylvestris* var. *mongolica* growth and nutrient assimilation, (3) mitigate heavy metal pollution risk for sandy soil. By quantifying the soil–plant feedback mechanisms under ternary amendments, this study will provide empirical evidence for optimizing organic waste valorization in dryland afforestation, thereby bridging the knowledge gap between ecological risk management and precision fertility enhancement for *Pinus sylvestris* var. *mongolica*.

## 2. Results

### 2.1. Effects on Soil pH, Moisture Content, and Organic Carbon

**Figure 1:**
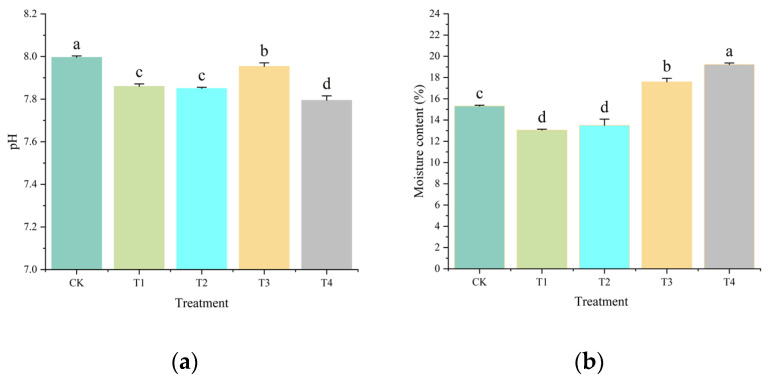
Effects of different organic amendments on soil pH (**a**) and moisture content (**b**). Lowercase letters indicate significant differences among different treatments at *p* < 0.05. Error bars denote the standard deviation (*n* = 5).

The initial pH of the sandy soil was alkaline (8.0), which decreased significantly (*p* < 0.05) following the application of amendments (Figure 1a, Table S1). Among the treatments, T4 (SS + CM + MS) resulted in the most substantial reduction in pH to 7.79, followed by T1 (7.86), T2 (7.85), and T3 (7.95), all of which were significantly lower than CK (8.0). Soil moisture content exhibited significant variation across the treatments (Figure 1b, Table S1). CK maintained moisture content of 15.31%, whereas T4 demonstrated the highest moisture content at 19.23%, representing a 25.60% increase over CK (*p* < 0.05). Conversely, T1 (13.05%) and T2 (13.50%) exhibited significantly lower moisture content compared to CK.

**Figure 2:**
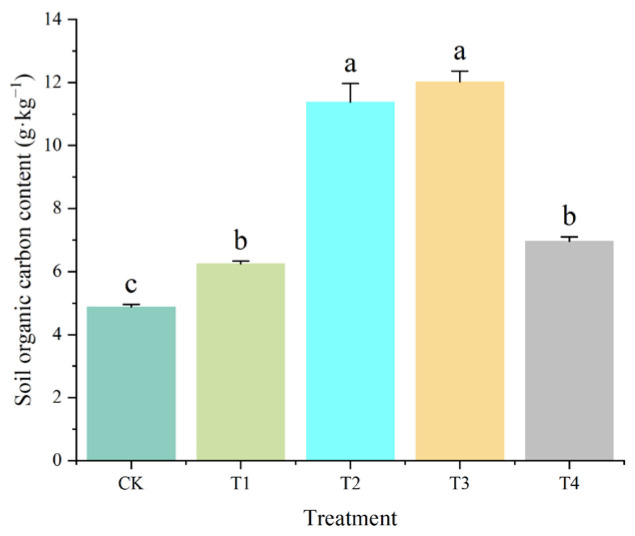
Effects of different organic amendments on SOC content. Lowercase letters indicate significant differences among different treatments at *p* < 0.05. Error bars denote the standard deviation (*n* = 5).

The application of amendments significantly enhanced soil organic carbon (SOC) contents (Figure 2, Table S1). The SOC content in CK was 4.88 g·kg^−1^, while T3 (11.38 g·kg^−1^) and T4 (12.02 g·kg^−1^) achieved increases of 2.3–fold and 2.5–fold, respectively (*p* < 0.05). T1 (6.25 g·kg^−1^) and T2 (6.96 g·kg^−1^) also demonstrated significant enrichment in SOC relative to CK.

### 2.2. Effects on Soil Total Nitrogen, Total Phosphorus, Ammonium Nitrogen, and Nitrate Nitrogen

**Figure 3:**
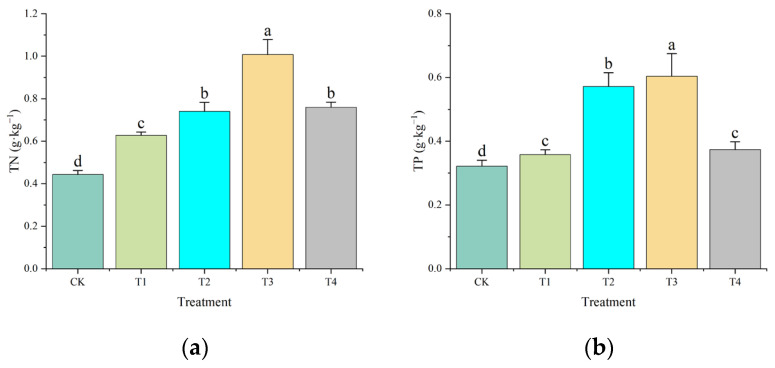
Effects of different organic amendments on contents of soil TN (**a**) and TP (**b**). Lowercase letters indicate significant differences among different treatments at *p* < 0.05. Error bars denote the standard deviation (*n* = 5).

The content of total nitrogen (TN) was significantly affected by the type of amendment applied, as illustrated in Figure 3a, Table S1. The TN content increased from 0.44 g·kg^−1^ in the control (CK) to 1.00 g·kg^−1^ in treatment T3, representing a 127% increase (*p* < 0.05). Treatments T2 and T4 resulted in moderate increases in TN content, measuring 0.74 g·kg^−1^ and 0.76 g·kg^−1^, respectively, while T1 exhibited the least effect with a TN content of 0.63 g·kg^−1^.

Regarding total phosphorus (TP), treatment T3 again demonstrated the most pronounced effect, achieving a TP content of 0.60 g·kg^−1^, which corresponds to an 87.5% increase over the control (0.32 g·kg^−1^; *p* < 0.05; Figure 3b, Table S1). Treatments T2 and T4 showed intermediate effects with TP contents of 0.57 g·kg^−1^ and 0.37 g·kg^−1^, respectively, whereas T1 resulted in minimal TP enhancement at 0.36 g·kg^−1^.

**Figure 4:**
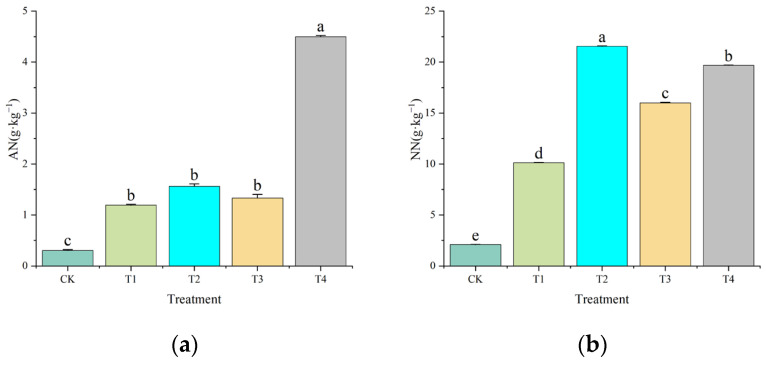
Effects of different organic amendments on contents of soil AN (**a**) and NN (**b**). Lowercase letters indicate significant differences among different treatments at *p* < 0.05. Error bars denote the standard deviation (*n* = 5).

The content of ammonium nitrogen (AN) responded variably to the amendments, as depicted in Figure 4a, Table S1. The AN content in CK was 0.30 g·kg^−1^, which increased following amendment application to 1.19 g·kg^−1^ (T1), 1.57 g·kg^−1^ (T2), 1.33 g·kg^−1^ (T3), and 4.50 g·kg^−1^ (T4). Notably, T4 led to a 14–fold increase in AN relative to CK (*p* < 0.05), suggesting synergistic effects of combined organic amendments.

The dynamics of nitrate nitrogen (NN) exhibited distinct patterns, as illustrated in Figure 4b, Table S1. The control soil contained 2.10 g·kg^−1^ of NN, whereas the application of amendments significantly increased these levels to 10.13 g·kg^−1^ (T1), 21.54 g·kg^−1^ (T2), 16.00 g·kg^−1^ (T3), and 19.68 g·kg^−1^ (T4). Notably, treatment T2 resulted in the highest NN enrichment, representing a 10.3-fold increase compared to the control (CK), and significantly surpassed the other treatments by 46.8–112.9% (*p* < 0.05).

### 2.3. Heavy Metal Concentrations in Amended Soils

Heavy metal concentrations (Cd, Cu, Pb, Zn) across treatments are summarized in Table 1 and Table S2. Compared to the control (CK: Cd 0.138 mg·kg^−1^, Cu 13.6 mg·kg^−1^, Pb 19.3 mg·kg^−1^, Zn 23.9 mg·kg^−1^), T4 (SS + CM + MS) exhibited the most significant reduction: Cd decreased by 54.6% (0.063 mg·kg^−1^), Cu by 35.1% (8.83 mg·kg^−1^), Pb by 12.2% (16.95 mg·kg^−1^), and Zn by 27.6% (17.3 mg·kg^−1^). Notably, all treatments maintained metal levels below China’s agricultural soil contamination risk thresholds (GB 15618-2018 [14]: Cd ≤ 0.6 mg·kg^−1^, Cu ≤ 100 mg·kg^−1^; Pb ≤ 170 mg·kg^−1^; Zn ≤ 300 mg·kg^−1^).

### 2.4. Effects on Plant Basal Diameter, Height and Biomass

The application of organic amendments markedly improved the morphological development of *Pinus sylvestris* var. *mongolica* in sandy soil, as presented in Table 2 and Table S3. In comparison to the control (CK), which had a basal diameter of 5.83 cm, treatments T1 (SS) and T2 (SS + CW) increased the basal diameter by 65.59% (9.66 cm) and 44.77% (8.44 cm), respectively (*p* < 0.05). A similar pattern of enhancement was observed in plant height, with T1 achieving the greatest growth (34.20 cm compared to CK’s 26.40 cm), representing a 29.50% increase (*p* < 0.05). Biomass accumulation in all amended treatments exceeded CK levels, thereby confirming the effectiveness of the amendments in promoting pine growth under sandy soil conditions.

### 2.5. Effects of Amendments on Nutrient Content in Plant Tissues

Post-amendment, significant changes in contents of total nitrogen (TN), total phosphorus (TP), and organic carbon (OC) were noted in *Pinus sylvestris* var. *mongolica* tissues (roots, branches, leaves) as shown in Table 3 and Table S3.

Root TN increased from 5.12 g·kg^−1^ (CK) to 6.80 (T1), 8.15 (T2), 6.77 (T3), and 6.12 g·kg^−1^ (T4), with T2 having the highest rise (59.20%, *p* < 0.05). Root TP rose from 0.68 g·kg^−1^ (CK) to 0.75 (T1), 1.08 (T2), and 0.81 g·kg^−1^ (T4), while T3 showed an 8.8% decrease compared to CK (*p* < 0.05). Root OC increased by 29.16% in T4 (73.26 g·kg^−1^ vs. CK 56.72 g·kg^−1^, *p* < 0.05).

Branch TN rose from 8.36 g·kg^−1^ (CK) to 9.07 (T1, +8.6%) and 9.25 (T2, +10.60%) g·kg^−1^ (*p* < 0.05). Branch TP peaked in T2 (1.51 g·kg^−1^, +24.50% vs. CK 1.21 g·kg^−1^, *p* < 0.05), while T1 (1.10 g·kg^−1^) and T4 (1.01 g·kg^−1^) decreased. Branch OC fell by 23.8% in T3 (53.42 g·kg^−1^ vs. CK 70.07 g·kg^−1^), whereas T1 saw a 0.37% increase.

Foliar total nitrogen (TN) contents exhibited a significant increase from 15.43 g·kg^−1^ in the control (CK) to 21.04 g·kg^−1^ in treatment T3, representing a 36.40% increase (*p* < 0.05). In contrast, foliar total phosphorus (TP) did not demonstrate significant differences among treatments, although treatment T2 achieved a 10.2% increase (1.82 g·kg^−1^ compared to CK: 1.65 g·kg^−1^). The response of foliar organic carbon (OC) varied among treatments: treatment T4 resulted in an 17.85% increase in OC (193.97 g·kg^−1^ compared to CK: 164.59 g·kg^−1^, *p* < 0.05), whereas treatments T2 and T3 showed decreases in OC by 13.25% and 9.87%, respectively.

## 3. Discussion

### 3.1. Effect of Amendment Application on Soil Properties

The initial alkaline pH (8.0) of the sandy soil was significantly decreased following the application of amendments, most notably in treatment T4 (SS + CM + MS; pH 7.79). This observation is consistent with global meta-analyses, which indicate that organic amendments, such as manure and straw, can reduce soil pH by up to 17% in acidic soils through mechanisms involving proton release and organic acid production [15]. In contrast, in alkaline soils, the reduction in pH is likely attributable to the microbial mineralization of organic matter, which releases CO_2_ and organic acids, as evidenced in sandy soils treated with poultry manure [16]. The observed pH reduction in T4 (SS + CM + MS) aligns with established mechanisms of organic acid release during microbial decomposition of complex organic substrates. Low-molecular-weight organic acids (e.g., citric, oxalic acids) produced by heterotrophic microorganisms can complex with soil minerals and protons, thereby lowering pH [17,18]. Notably, the combined addition of CM and MS likely enhanced fungal activity, as lignocellulose–rich straw stimulates cellulolytic fungi capable of releasing organic acids via glycolytic pathways [19]. Such acidification processes have been documented in sludge amended soils, where organic acid accumulation correlates with microbial biomass shifts toward acid tolerant taxa.

Furthermore, the 25.6% increase in soil moisture retention under T4 (Figure 1b) aligns with established mechanisms, whereby organic amendments reduce bulk density and increase porosity [20,21]. A meta–analysis of urban and agricultural soils has demonstrated that organic amendments, such as compost and biochar, can increase soil water-holding capacity by 10–25% through improved aggregate stability and reduced bulk density [22]. Similar patterns were observed in aeolian sandy soils amended with corn straw and chicken manure, where organic amendments enhanced water content by 25–30% [23]. The observed reduction in moisture content in treatments T1 and T2 may be attributed to the predominance of sewage sludge (SS), which could increase soil hydrophobicity or alter pore size distribution [24].

Integrated amendments (SS+CM+MS) synergistically modulated soil organic carbon (SOC) stabilization and nutrient cycling through microbial–mediated processes. The 2.5–fold SOC increase in T4 (Figure 2) aligns with lignin–cellulose decomposition enhancing particulate organic matter (POM), while humic acid–metal complexation promoted mineral-associated organic carbon (MAOC) formation [25]. Concurrently, TN and TP dynamics were driven by shifts in functional guilds: nitrogen-fixing *Bradyrhizobium* and phosphorus-solubilizing Streptomyces dominated rhizospheres in T2/T4, consistent with studies showing manure–straw blends elevate *β-glucosidase* and *phosphatase* activities by 40–60% [26,27]. These enzymes catalyzed SOM mineralization, releasing bioavailable N and P while stabilizing carbon via microbial necromass accumulation—a process contributing >25% of SOC in amended sandy soils [28].

The divergent responses of ammonium (AN) and nitrate (NN) nitrogen (Figure 4a,b) suggest microbial-mediated nitrogen transformations. The 14-fold AN accumulation in T4 may result from temporary suppression of nitrifier communities (e.g., *Nitrosomonas* and *Nitrobacter*) due to acidic pH (<7.8) and elevated organic carbon, which promotes heterotrophic dominance over autotrophic nitrifiers [29,30]. Conversely, the elevated NN in T2 (SS + CM) indicates nitrifier stimulation, potentially driven by moderate pH (7.85) and enhanced aeration from CM induced soil aggregation. These findings align with studies demonstrating that biochar–organic blends selectively enrich Nitrospira by adsorbing inhibitory phenolic compounds [29]. Research conducted in subtropical forests has demonstrated that the application of biochar amendments mitigates microbial nitrogen limitation by enhancing substrate availability, potentially explaining the balanced AN/NN ratios observed in treatments T3 and T4 [31]. Furthermore, the synergistic effects of combined amendments, such as gypsum and straw, in coastal saline soils have been shown to facilitate sodium displacement and increase calcium availability, thereby indirectly promoting nitrogen stabilization [32]. These mechanisms corroborate our hypothesis that integrated amendments optimize nitrogen cycling by modulating both physicochemical and biological processes.

Post-amendment, significant changes in total nitrogen (TN), total phosphorus (TP), and organic carbon (OC), were noted in *Pinus sylvestris* tissues (roots, branches, leaves) as shown in Table 2.

### 3.2. Influence of Amendments on the Growth of Pinus sylvestris var. mongolica

The application of sewage sludge (SS) as a soil amendment significantly enhances the growth of *Pinus sylvestris* var. *mongolica* in degraded sandy ecosystems. Field studies by Bai et al. [33] demonstrated that SS application (25 t ha^−1^) increased height by 25% and basal diameter by 47.87% compared to unamended controls, highlighting its potential for rehabilitating nutrient-poor soils. In our pot-based experiment, the synergistic integration of sewage sludge (SS), cattle manure (CM), and maize straw (MS) in treatment T4 significantly enhanced *Pinus sylvestris* var. *mongolica* growth and soil nutrient dynamics in degraded sandy soils. The ternary amendment achieved a 29.5% increase in plant height and 65.6% greater basal diameter compared to the control (CK), aligning with findings by Bai et al. [33] in similar semi-arid ecosystems. While the pot experiment faced limitations in simulating natural field conditions (e.g., restricted root zone dynamics), significant growth responses were observed even in 3-year-old seedlings. These results suggest that early-stage *Pinus sylvestris* var. *mongolica* is highly responsive to organic amendments. These improvements were mechanistically linked to enhanced soil porosity and microbial-driven nutrient cycling, as SS-derived organic matter facilitated hyphal network formation by lignocellulose-degrading *Ascomycota* fungi [34], while CM supplied humic acid-stabilized metal pollutants (Cd: −54.6%, Cu: −35.1%, Pb: −12.2% and Zn: −27.6%) through chelation and redox reactions [35,36]. The positive effects of organic amendments on plant growth and biomass in sandy soils are corroborated by the findings of Gul et al., Thongchai et al., and Wiafe et al. [37,38,39].

The application of soil amendments in sandy soil resulted in significant variations in TN, TP, and OC content within the tissues of Mongolian pine, as presented in Table 2. These findings are consistent with recent research on nutrient dynamics influenced by microbial activity and rhizosphere interactions. For example, the observed increase in TN in the roots of plant under the T2 treatment (a 59.2% increase) may be attributed to enhanced nitrogen fixation and nitrification processes facilitated by soil microbial communities. Hu et al. [40] demonstrated that conservation tillage practices, such as reduced tillage, significantly increased the relative abundance of nitrogen-fixing bacteria (e.g., *Mesorhizobium* sp., *Bradyrhizobium* sp.) and nitrifiers (e.g., *Nitrosospira* sp.), which could synergistically enhance nitrogen availability in plant roots. This mechanism likely contributed to the observed accumulation of TN in T2-treated roots, particularly under the nutrient-limited conditions of sandy soil.

The varied TP responses observed across different treatments, such as the 24.5% increase in branches under T2 and the 8.8% decrease in roots under T3 which underscored the intricate relationship between phosphorus availability and microbial-mediated nutrient cycling. Lu et al. [41] demonstrated that phosphorus fertilization can influence rhizosphere priming effects (RPE) by modifying microbial nitrogen immobilization, thereby indirectly impacting phosphorus uptake efficiency in a manner contingent on plant species and root characteristics. In our investigation, the elevated TP levels in T2 branches may be attributed to enhanced phosphorus solubilization facilitated by microbial exudates. Conversely, the reduction in TP in T3 roots could be due to competitive microbial phosphorus uptake under conditions of carbon-rich amendments, a phenomenon previously observed in halophyte-associated microbiomes where such amendments prompted microbial phosphorus scavenging to satisfy stoichiometric requirements [42].

The observed treatment-dependent variations in organic carbon (OC), notably the 29.16% increase in T4 roots, indicate that the application of combined amendments, such as organic and microbial inputs, enhances carbon sequestration through the stabilization of microbial networks. Recent studies have highlighted that conservation tillage supports stable microbial ecological networks, predominantly composed of keystone taxa like *Burkholderia* sp., which contribute to the stabilization of organic carbon through the production of extracellular enzymes and a reduction in decomposition rates [43]. This observation is consistent with our findings that the T4 treatment, involving a triple amendment, achieved maximum OC accumulation, potentially by fostering cooperative microbial interactions.

In the foliage, the 36.4% increase in TN under the T3 treatment highlights the significance of nitrogen translocation from roots to shoots under conditions of nutrient stress. This phenomenon aligns with the findings of Jing et al. [44], which demonstrated that nitrogen addition (0, 3, 6, and 9 g N m^−2^ y^−1^) in *Pinus tabuliformis* forests enhances foliar nitrogen assimilation while suppressing nitrous oxide emissions, indicating a trade-off between plant nitrogen uptake and microbial nitrogen transformation processes. Similarly, the non-significant variations in TP in foliage, despite a 10.2% increase in T2, may reflect limitations in phosphorus redistribution. This is akin to observations in legume–grass systems, where phosphorus fertilization preferentially enhances root TP over shoot TP due to stoichiometric constraints within the rhizosphere [41]. Furthermore, the 17.85% increase in foliar OC in T4 is likely attributable to enhanced photochemical efficiency and microbial priming resulting from amendments. Recently demonstrated that halophyte-associated archaea (*Halobacteria*) in arid soils increase carbon-use efficiency by 34% through optimized glycolysis pathways, a phenomenon that may be applicable to sandy soil microbiomes under organic-inorganic amendment blends [42]. This, in conjunction with lignin deposition induced by microbial volatile organic compounds (mVOC_s_), establishes a positive feedback loop conducive to long-term carbon sequestration [45].

### 3.3. Implications for Pollution Risk Mitigation Mechanism via Partial Substitution Means

The ternary amendment (SS + CM + MS) synergistically reduces sludge-derived risks via three interconnected pathways. Firstly, the dilution effect from partial sludge substitution (40% in T4) inherently lowers total heavy metal inputs. For instance, bioavailable Cd decreased by 54.6% in T4 compared to sludge-only treatments (Table 3), consistent with studies showing that co-application of organic amendments (e.g., straw, manure) dilutes contaminants by increasing soil organic matter and promoting metal immobilization through lignocellulose adsorption and humic acid complexation [46,47]. Yang et al. [47] demonstrated that biochar derived from sludge and straw co-pyrolysis reduces Cd bioavailability by 32–48% in sandy soils through similar mechanisms. Secondly, microbial suppression is driven by thermophilic bacteria in CM and lignocellulose-degrading fungi in MS. CM elevates decomposition temperatures (>55 °C), inhibiting mesophilic pathogens (e.g., *E. coli*), while MS fosters *Streptomyces* and *Trichoderma* fungi that secrete lytic enzymes (e.g., chitinases) to degrade pathogen cell walls [48,49,50]. Wang et al. [49] reported that manure–straw mixtures reduce Salmonella survival by 90% in agricultural soils through competitive exclusion and enzymatic antibiosis. Thirdly, humic acid–metal complexation stabilizes heavy metals. Humic acids from CM and MS form chelates with Cd and Zn, reducing their phytoavailability, as evidenced by a 35% decline in Zn mobility in T4 soils (Table 3). This aligns with Agnieszka et al. [51], who found that humic acid complexes decrease Cd bioavailability. Collectively, these mechanisms underscore the ecological safety of integrated amendments in balancing soil fertility and contaminant control.

Synergistic mechanisms (e.g., *lignocellulose* degradation, microbial suppression) are informed by soil chemistry and plant growth data, direct microbial community profiling (e.g., 16S rRNA sequencing, phospholipid fatty acid analysis) and enzyme activity (e.g., phosphatase, dehydrogenase) have been demonstrated by several researchers. For instance, Zhou et al. [52] demonstrated that co-incorporating maize straw and manure increased *β-glucosidase* and cellulase activities by 40–60%, directly linking microbial functional shifts to carbon release. Similarly, Gajda et al. [53] used DGGE analysis to confirm *Actinobacteria* dominance in manure-amended soils, aligning with our hypothesis of lignocellulose-degrading fungi enrichment. We recommend quantifying microbial biomass carbon (MBC) and nitrogen (MBN) via chloroform fumigation extraction, as demonstrated by Liang et al. [34] in sludge-amended soils, to resolve microbial contributions to nutrient turnover. Additionally, enzyme kinetics assays (e.g., *urease*, *arylsulfatase*) could validate organic acid-mediated pH reduction pathways [53,54]. Future investigations should integrate metagenomics and stable isotope probing (SIP) to resolve taxa-specific contributions to nutrient cycling.

## 4. Materials and Methods

### 4.1. Study Site Description

The study was conducted at Red Flag Township, Xinmin City in Liaoning Province, which is a typical semiarid area (122°33′40′′ E, 41°51′53′′ N; about 29 m above sea level). The study area experienced a temperate continental monsoon, with a mean annual temperature of 8.2 °C, a frost-free period of 160 days, and an average wind speed of 4.1 m·s^−1^ [55]. The mean annual precipitation is about 417.7 mm, with over 60% of rainfall occurring between July and September. Mean annual sunshine duration is 2753.2 h and active accumulated temperature is 3348 °C. The soil is weak alkaline (7.5–8.0) and deficient in organic matter, nitrogen, and phosphorus.

### 4.2. Soil, Amendments, and Seedlings Preparation

Soil samples were collected at a location around Red Flag Township from the 0–20 cm soil layer in 2024. These soils are characterized by low nutrients. In this study, soil amendments were organic wastes (sewage sludge, SS; cattle manure, CM; and maize straw, MS). The properties of the soil, SS, CM, and MS are shown in Table 4. There were five treatments, with five replications per treatment: control with no amendment addition (CK), SS (T1), SS + CM (T2), SS + MS (T3), and SS + CM + MS (T4). For each treatment, 5 seedling-raising pots with a height of 30 cm and a diameter of 30 cm were prepared. Each pot was filled with 30.0 kg of soils. The organic amendments were applied into the soil at a proportion of 1:100 (amendments: soil, w:w) and mixed well (Table 5). The 1:100 amendment-to-soil ratio was selected based on previous studies in semi-arid ecosystems [3,33], which demonstrated optimal nutrient release with minimal salinity risks. Three-year-old nursery-raised seedlings of *Pinus sylvestris* var. *mongolica* were selected for uniform growth and transplanted individually into pots. Therefore, a total of 25 pots were employed for this experiment (five amendments × five replicates). Pots were arranged in a completely randomized design to assign treatments to grid positions, minimizing microenvironmental bias. After transplanting, equal amounts of water were used in each pot to imitate the field condition. Seedlings were then left to grow under natural conditions in the field until the end of the experiment. Similar conditions were provided for the experimental pots. For example, weeds under these seedlings were removed regularly. The period of our experiment was from May to October 2024. During the experimental period, the total amount of precipitation was 797.5 mm. The average daily air temperature and relative humidity were 21.3 °C and 82.9%, respectively.

### 4.3. Seedling Survey, Soil Sampling and Chemical Analysis

At the end of the experiment, the height and basal diameter of all seedlings were measured using a steel tape and caliper, respectively. The seedlings were then harvested, carefully washed with deionized water, and separated into leaves, stems, and roots. The dry biomass of each plant component was determined after oven-drying the samples at 65 °C for 72 h. The total biomass production (aboveground and belowground) and the aboveground/belowground ratio of plant were calculated for each treatment. Samples of the different plant components were ground using a ball mill and passed through a 0.15 mm sieve for the determination of OC, TN, and TP.

Soil samples from each pot treated with amendments were collected for physicochemical analyses. For fresh soil samples, soil moisture was determined by oven-drying at 105 °C until a constant weight was achieved. Soil inorganic nitrogen (NO_3_-N and NH_4_^+^–N) was extracted using a 2 mol·L^−1^ KCl solution and analyzed using an Auto Analyzer III. The air-dried portion of the soil was used to analyze soil physicochemical properties. Soil pH was measured in a soil–water suspension (10 g soil and 25 mL distilled water) using a pH meter. OC, TN, and TP were measured after the air-dried soil was ground to pass through a 0.25 mm sieve. The OC content of the air-dried soil and plant samples was analyzed using the K_2_Cr_2_O_7_–H_2_SO_4_ oxidation method. TN and TP in the soil and plant samples were determined using an Auto Analyzer III after high-temperature digestion with H_2_SO_4_–CuSO_4_. Soil samples were digested using the HNO_3_–HClO_4_–HF method. Heavy metal concentrations (Pb, Cu, Zn) were determined using inductively coupled plasma mass spectrometry. Cd was determined using graphite furnace atomic absorption spectrophotometry.

### 4.4. Statistical Analysis

The data were processed and organized using Microsoft Excel 2024. Mean values were calculated from five replicates. To evaluate the effects of the amendments on seedling growth and soil properties, a one-way analysis of variance (ANOVA) was performed, followed by Tukey’s honestly significant difference (HSD) test for multiple comparisons among treatments. Prior to analysis, data were tested for normality and homogeneity of variances to determine whether the transformations were necessary before data analyses. Pearson’s correlation analysis was conducted to quantify the strength of linear relationships between soil nutrient status and plant growth parameters, as well as nutrient content in different plant components. Significance levels were denoted by * *p* < 0.05 and ** *p* < 0.01. All statistical analyses were performed using SPSS (Version 27.0, IBM Corp., Chicago, IL, USA).

## 5. Conclusions

This study demonstrates that the ternary amendment combining sewage sludge (SS), cattle manure (CM), and maize straw (MS) (T4) achieves synergistic effects in rehabilitating degraded sandy soils of the Horqin Sandy Land and enhancing *Pinus sylvestris* var. *mongolica* growth. The integrated amendment effectively reduced soil pH from 8.02 to 7.79, elevated organic carbon by 2.5-fold, and significantly increased total nitrogen (127%) and phosphorus (87.5%) compared to the control. These physicochemical improvements were accompanied by enhanced plant growth, with basal diameter and biomass accumulation in *Pinus sylvestris* increasing by 65.6% and 29.5%, respectively, alongside elevated foliar nitrogen assimilation (36.4% in T3). Crucially, T4 mitigated risks associated with sludge overuse by reducing heavy metal concentration (e.g., Cd by 54.6%, Cu by 35.1%, Pb by 12.2%, and Zn by 27.6%). These results underscore a circular economy approach that converts organic waste into ecological assets, balancing soil fertility enhancement with long-term safety. This work provides a sustainable framework for integrating waste valorization with dryland afforestation, advancing both ecological restoration and precision soil management. Future studies should prioritize multi-temporal monitoring of soil microbiome dynamics and nutrient flux to optimize amendment strategies for scaling in arid ecosystems.

## Figures and Tables

**Table 1:** Heavy metal concentrations in soils after amendment.

Heavy Metal Concentration (mg·kg^−1^)	CK	T1	T2	T3	T4
Cd	0.138 ± 0.14 a	0.091 ± 0.08 c	0.101 ± 0.11 b	0.105 ± 0.10 bc	0.063 ± 0.06 d
Cu	13.60 ± 0.16 a	12.50 ± 0.16 b	9.82 ± 0.01 d	11.70 ± 0.01 c	8.83 ± 0.01 e
Pb	19.30 ± 0.01 b	18.20 ± 0.01 d	19.90 ± 0.02 a	19.20 ± 0.01 c	16.95 ± 0.01 e
Zn	23.90 ± 0.01 a	21.10 ± 0.07 b	18.20 ± 0.01 d	20.90 ± 0.08 c	17.30 ± 0.01 e

Data are means ± SD (*n* = 5). Lowercase letters indicate significant differences among different treatments at *p* < 0.05.

**Table 2:** Effects of different organic amendments on plant basal diameter, height, and biomass.

Parameter	CK	T1	T2	T3	T4
Basal diameter (cm)	5.83 ± 0.34 b	8.44 ± 0.44 a	9.66 ± 2.38 a	7.80 ± 1.47 ab	7.74 ± 1.21 ab
Height (cm)	26.40 ± 4.62 b	34.20 ± 2.95 a	32.20 ± 4.82 ab	31.00 ± 2.65 ab	29.80 ± 4.02 ab
Biomass (g)	40.97 ± 3.90 b	56.15 ± 6.66 a	53.49 ± 4.45 a	52.84 ± 7.40 a	49.96 ± 5.71 a

Data are means ± SD (*n* = 5). Lowercase letters indicate significant differences among different treatments at *p* < 0.05.

**Table 3:** Effects of different organic amendments on TN, TP, and OC in various plant tissues.

Parameter	CK	T1	T2	T3	T4
TN in the roots (g·kg^−1^)	5.12 ± 0.24 c	6.79 ± 0.36 b	8.15 ± 1.04 a	6.77 ± 1.74 b	6.12 ± 0.41 bc
TN in the branches (g·kg^−1^)	8.36 ± 0.42 b	9.07 ± 0.47 a	9.25 ± 0.77 a	7.80 ± 0.25 b	7.89 ± 0.35 b
TN in the leaves (g·kg^−1^)	15.43 ± 1.364 c	17.01 ± 0.35 b	17.95 ± 1.74 b	21.04 ± 1.57 a	17.66 ± 0.58 b
TP in the root (g·kg^−1^)	0.68 ± 0.06 cd	0.75 ± 0.02 bc	1.08 ± 0.15 a	0.62 ± 0.10 d	0.81 ± 0.06 b
TP in the branches (g·kg^−1^)	1.21 ± 0.10 b	1.10 ± 0.06 bc	1.51 ± 0.30 a	1.07 ± 0.05 bc	1.01 ± 0.05 c
TP in leaves (g·kg^−1^)	1.65 ± 0.17 a	1.70 ± 0.07 b	1.82 ± 0.14 b	1.78 ± 0.24 b	1.73 ± 0.04 b
OC in the root (g·kg^−1^)	56.72 ± 1.69 b	51.97 ± 0.72 c	71.96 ± 2.84 a	70.70 ± 3.90 a	73.26 ± 0.61 a
OC in branches (g·kg^−1^)	70.07 ± 0.59 a	70.33 ± 1.60 a	67.96 ± 2.82 a	53.42 ± 2.39 c	57.72 ± 6.14 b
OC in the leaves (g·kg^−1^)	164.59 ± 2.38 c	168.05 ± 2.50 b	142.77 ± 1.90 e	148.34 ± 2.50 d	193.97 ± 3.36 a

Data are means ± SD (*n* = 5). Lowercase letters indicate significant differences among different treatments at *p* < 0.05.

**Table 4:** The properties of the soil, SS, CM, and MS.

Parameter	Soil	SS	CM	MS
pH	7.86 ± 0.04	6.74 ± 0.03	7.95 ± 0.03	-
Moisture content (%)	17.23 ± 0.45	10.51 ± 0.10	5.77 ± 0.36	4.29 ± 0.30
TN (g·kg^−1^)	0.44 ± 0.01	39.32 ± 1.58	11.44 ± 2.86	5.05 ± 0.19
TP (g·kg^−1^)	0.26 ± 0.01	10.74 ± 0.38	2.58 ± 0.55	0.51 ± 0.00
OC (g·kg^−1^)	4.30 ± 0.35	317.46 ± 14.53	374.73 ± 17.67	495.87 ± 21.80
Cu (mg·kg^−1^)	11.90 ± 0.04	105.00 ± 0.49	19.30 ± 0.01	9.86 ± 0.02
Cd (mg·kg^−1^)	0.10 ± 0.01	0.69 ± 0.01	0.12 ± 0.02	0.22 ± 0.03
Pb (mg·kg^−1^)	20.60 ± 0.01	29.00 ± 0.03	5.43 ± 0.01	3.06 ± 0.01
Zn (mg·kg^−1^)	17.70 ± 0.07	570.00 ± 0.71	43.60 ± 0.07	7.25 ± 0.01

SS, sewage sludge; CM, cattle manure; MS, maize straw.

**Table 5:** Treatment marks and types of organic amendments used in this study.

Treatments	Compositions	Source and Preparing of Organic Amendments
CK	Soil without amendment	The SS is consistent with those described by Bai et al. [33]. The CM and the MS were collected from a farm located near the Red Flag Township in October 2023, air-dried, and ground to pass through a 2 mm sieve.
T1	30.0 kg soil + 0.3 kg SS	
T2	30.0 kg soil + 0.15 kg SS + 0.15 kg CM	
T3	30 kg soil + 0.15 kg SS + 0.15 kg MS	
T4	30 kg soil + 0.10 kg SS + 0.10 kg CM + 0.10 kg MS	

SS, sewage sludge; CM, cattle manure; MS, maize straw.

## Data Availability

The data are contained in the article.

## Supplementary Materials

The following supporting information can be downloaded at: https://www.mdpi.com/article/10.3390/plants14132067/s1, Table S1: ANOVA on pH, Moisture Content, Organic Carbon, Total Nitrogen, Total Phosphorus, Ammonium Nitrogen and Nitrate Nitrogen in soil under different treatments; Table S2: ANOVA on heavy metal concentrations in soils under different treatments; Table S3: ANOVA on plant basal diameter, height and biomass under different treatments; Table S4: ANOVA on TN, TP and OC in various plant tissues under different treatments.
