# Supplementary material for: Synergistic Effects of Partial Substitution of Sludge with Cattle Manure and Straw on Soil Improvement and Pinus sylvestris var. mongolica Growth in Horqin Sandy Land, China
Source: Plants (Basel). 2025 Jul 6;14(13):2067. doi: 10.3390/plants14132067 (PMC12251849; doi:10.3390/plants14132067)
Supplement: Supplementary file 1 [file plants-14-02067-s001.zip › plants-3702295-supplementary.pdf]

Table S1. ANOVA on pH,Moisture Content,Organic Carbon,Total Nitrogen,Total Phosphorus,Ammonium Nitrogen and Nitrate Nitrogen in soil under different treatments.

Parameter	ANOVA						
				df	MS	*F value*	*P value*
pH	Between Groups	(Combination)		4	0.034	36.041	0.000
		Linear TermL	Contrast	1	0.048	51.226	0.000
			Deviation	3	0.029	30.979	0.000
	Within Groups			20	0.001		
	Total			24			
				df	MS	*F value*	*P value*
Moisture Content (%)	Between Groups	(Combination)		4	35.068	60.776	0.000
		Linear TermL	Contrast	1	76.558	132.684	0.000
			Weighted	3	21.238	36.807	0.000
	Within Groups			20	0.577		
	Total			24			
				df	MS	*F value*	*P value*
Organic Carbon ($g\cdot kg^{-1}$)	Between Groups	(Combination)		4	51.306	104.995	0.000
		Linear TermL	Contrast	1	49.342	100.976	0.000
			Weighted	3	51.961	106.335	0.000
	Within Groups			20	0.489		
	Total			24			
				df	MS	*F value*	*P value*
Total Nitrogen ($g\cdot kg^{-1}$)	Between Groups	(Combination)		4	0.212	26.538	0.000
		Linear TermL	Contrast	1	0.512	64.137	0.000
			Weighted	3	0.112	14.005	0.000
	Within Groups			20	0.008		
	Total			24			

				df	MS	*F value*	*P value*
Total Phosphorus (g·kg^{-1})	Between Groups	(Combination)		4	0.086	134.208	0.000
		Linear TermL	Contrast	1	0.061	95.109	0.000
			Weighted	3	0.095	147.241	0.000
		Within Groups		20	0.001		
		Total		24			
				df	MS	*F value*	*P value*
Ammonium Nitrogen (g·kg^{-1})	Between Groups	(Combination)		4	12.706	122.348	0.000
		Linear TermL	Contrast	1	36.398	350.468	0.000
			Weighted	3	4.809	46.308	0.000
		Within Groups		20	0.104		
		Total		24			
				df	MS	*F value*	*P value*
Nitrate Nitrogen (g·kg^{-1})	Between Groups	(Combination)		4	312.174	273.429	0.000
		Linear TermL	Contrast	1	841.977	737.475	0.000
			Weighted	3	135.574	118.747	0.000
		Within Groups		20	1.142		
		Total		24			

Table S2. ANOVA on heavy metal concentrations in soils under different treatments.

Parameter	ANOVA						
				df	MS	*F value*	*P value*
Cd ($mg \cdot kg^{-1}$)	Between Groups	(Combination)		4	0.004	31.166	0.000
		Linear TermL	Contrast	1	0.010	77.300	0.000
			Deviation	3	0.002	15.789	0.000
	Within Groups			20	0.000		
	Total			24			
				df	MS	*F value*	*P value*
Cu ($mg \cdot kg^{-1}$)	Between Groups	(Combination)		4	18.969	1887.254	0.000
		Linear TermL	Contrast	1	53.437	5316.460	0.000
			Weighted	3	7.480	744.186	0.000
	Within Groups			20	0.010		
	Total			24			
				df	MS	*F value*	*P value*
Pb ($mg \cdot kg^{-1}$)	Between Groups	(Combination)		4	6.697	63821.253	0.000
		Linear TermL	Contrast	1	6.830	65086.792	0.000
			Weighted	3	6.653	63399.407	0.000
	Within Groups			20	0.000		
	Total			24			
				df	MS	*F value*	*P value*
Zn ($mg \cdot kg^{-1}$)	Between Groups	(Combination)		4	34.146	12687.156	0.000
		Linear TermL	Contrast	1	90.734	33712.286	0.000
			Weighted	3	15.284	5678.80	0.000
	Within Groups			20	0.003		
	Total			24			

Table S3. ANOVA on plant basal diameter, height and biomass under different treatments.

Parameter	ANOVA						
				df	MS	*F value*	*P value*
Biomass (g)	Between Groups	(Combination)		4	145.403	4.559	.011
		Linear TermL	Unweighted	1	92.320	2.895	.107
			Weighted	1	56.003	1.756	.203
			Deviation	3	175.203	5.494	.008
		Within Groups		17	31.892		
		Total		21			
				df	MS	*F value*	*P value*
Basal diameter (cm)	Between Groups	(Combination)		4	8.532	3.993	0.018
		Linear TermL	Unweighted	1	4.361	2.041	0.171
			Weighted	1	3.273	1.532	0.233
			Deviation	3	10.284	4.813	0.013
		Within Groups		17	2.137		
		Total		21			
				df	MS	*F value*	*P value*
Height (cm)	Between Groups	(Combination)		4	42.317	2.612	0.070
		Linear TermL	Unweighted	1	6.075	0.375	0.548
			Weighted	1	6.324	0.390	0.540
			Deviation	3	54.315	3.353	0.042
		Within Groups		18	16.200		
		Total		22			

Table S4. ANOVA on TN, TP and OC in various plant tissues under different treatments.

Parameter	ANOVA						
				df	MS	*F value*	*P value*
TN in the roots (g·kg^{-1})	Between Groups	(Combination)		4	6.124	6.877	0.001
		Linear TermL	Contrast	1	1.964	2.206	0.153
			Deviation	3	7.511	8.434	0.001
		Within Groups		20	0.891		
		Total		24			
				df	MS	*F value*	*P value*
TN in the branches (g·kg^{-1})	Between Groups	(Combination)		4	2.212	9.399	0.000
		Linear TermL	Contrast	1	2.455	10.431	0.004
			Weighted	3	2.131	9.055	0.001
		Within Groups		20	0.235		
		Total		24			
				df	MS	*F value*	*P value*
TN in the leaves (g·kg^{-1})	Between Groups	(Combination)		4	21.029	13.449	0.000
		Linear TermL	Contrast	1	36.142	23.115	0.000
			Weighted	3	15.991	10.227	0.000
		Within Groups		20	1.564		
		Total		24			
				df	MS	*F value*	*P value*
TP in the root (g·kg^{-1})	Between Groups	(Combination)		4	0.156	19.343	0.000
		Linear TermL	Contrast	1	0.008	0.953	0.341
			Weighted	3	0.206	25.472	0.000
		Within Groups		20	0.008		
		Total		24			
				df	MS	*F value*	*P value*
TP in the branches (g·kg^{-1})	Between Groups	(Combination)		4	0.192	8.749	0.000

		Linear TermL	Contrast	1	0.091	4.132	0.056
			Weighted	3	0.226	10.288	0.000
		Within Groups		20	0.022		
		Total		24			
				df	MS	*F value*	*P value*
TP in the leaves (g·kg-1)	Between Groups	(Combination)		4	0.022	0.966	0.448
		Linear TermL	Contrast	1	0.031	1.401	0.250
			Weighted	3	0.018	0.821	0.497
		Within Groups		20	0.022		
		Total		24			
				df	MS	*F value*	*P value*
OC in the root $(g\cdot kg^{-1})$	Between Groups	(Combination)		4	484.316	59.707	0.000
		Linear TermL	Contrast	1	1341.827	165.421	0.000
			Weighted	3	198.480	24.469	0.000
		Within Groups		20	8.112		
		Total		24			
				df	MS	*F value*	*P value*
OC in the branches $(g\cdot kg^{-1})$	Between Groups	(Combination)		4	304.756	28.068	0.000
		Linear TermL	Contrast	1	865.446	79.706	0.000
			Weighted	3	117.859	10.855	0.000
		Within Groups		20	10.858		
		Total		24			
				df	MS	*F value*	*P value*
OC in the leaves $(g\cdot kg^{-1})$	Between Groups	(Combination)		4	2011.900	303.911	0.000
		Linear TermL	Contrast	1	762.373	115.162	0.000
			Weighted	3	2428.409	366.827	0.000
		Within Groups		20	6.620		
		Total		24			
